# The efficacy of temozolomide combined with levetiracetam for glioblastoma (GBM) after surgery: a study protocol for a double-blinded and randomized controlled trial

**DOI:** 10.1186/s13063-022-06168-1

**Published:** 2022-03-28

**Authors:** Maoyuan Sun, Ning Huang, Yihao Tao, Rong Wen, Guanjian Zhao, Xiang Zhang, Zongyi Xie, Yuan Cheng, Jinning Mao, Guodong Liu

**Affiliations:** 1 Department of Neurosurgery, The Second Affiliated Hospital of Chongqing Medical University, Chongqing, 400000 China; 2 Department of Cardiology, The Second Affiliated Hospital of Chongqing Medical University, Chongqing, 400000 China; 3 Experimental Therapeutics and Molecular Imaging Laboratory, Department of Neurology, Neuro-Oncology Division, Massachusetts General Hospital, Boston, MA 02114 USA; 4 Neuroscience Program, Harvard Medical School, Boston, MA 02114 USA

**Keywords:** Glioblastoma (GBM), Temozolomide, Levetiracetam, Chemotherapy, Clinical trial

## Abstract

**Background:**

Temozolomide is applied as the standard chemotherapy agent in patients with glioblastoma (GBM) after surgery. However, the benefit of this treatment for patients is limited by the invasive growth of gliomas and drug resistance. There are indications from fundamental experimental and retrospective studies that levetiracetam has the potential to improve the survival rate of patients with GBM. However, it has yet to be determined whether the combination of temozolomide and levetiracetam is more effective than standard temozolomide chemotherapy. Therefore, we designed a randomized clinical trial to investigate the therapeutic effect of the new combined regime for treating GBM.

**Methods/design:**

This is a double-blind and randomized clinical trial conducted in a single center. One hundred forty-two patients will be recruited and screened for the inclusion and exclusion criteria. Then, eligible participants will be randomly assigned to an experimental group or a control group in a 1:1 ratio. Based on the administration of radiation therapy (RT), participants in the experimental group will be prescribed levetiracetam plus temozolomide chemotherapy for 34 weeks while participants in the control group will receive placebo tablets plus temozolomide for the same duration. A 3-year follow-up will be conducted on all patients after intervention. Accordingly, the primary outcome will be progression-free survival (PFS). The secondary endpoints include overall survival (OS), the Karnofsky Performance Status (KPS), the objective response rate (ORR), and adverse event incidence.

**Discussion:**

It is expected that the results of this trial will provide high-level evidence regarding the clinical benefits of levetiracetam and temozolomide combined in the treatment of GBM.

**Trial registration:**

Chinese Clinical Trial Registry, ChiCTR2100049941. Registered on 14 August 2021

**Supplementary Information:**

The online version contains supplementary material available at 10.1186/s13063-022-06168-1.

## Background

Glioblastoma (GBM) accounts for up to 60% of all primary malignant brain tumors in adults [1] and it is also known as astrocytoma grade IV according to 2016 WHO classification [2]. GBM is the most aggressive and lethal form of primary astrocytoma, due to the highly infiltrative and heterogeneous nature of the glioma cells. Currently, the standard of care for GBM patients consists of maximal surgical resection plus adjuvant chemotherapy and radiation therapy (RT) [3]. Despite multimodal treatment, the prognosis of patients with GBM is still extremely poor with a 5-year overall survival rate of less than 5% post-diagnosis [4]; 2–3% of patients survive for up to 2 years [5].

Of the many chemotherapy agents that are clinically available, temozolomide (TMZ) is the only standard chemotherapy available to patients with GBM [6, 7]. TMZ is an oral alkylating agent that penetrates the brain and damages DNA by inducing DNA O^6^-methylguanine [8]. However, the expression of the DNA repair enzyme O^6^-methylguanine methyltransferase (MGMT) can abrogate the cytotoxic O^6^-methylguanine DNA adduct by repairing damaged DNA and thus contribute to drug resistance [9]. In addition, the overexpression of the ATP-binding cassette (ABC) efflux transporters, ABCG2 and ABCB1, provides chemoresistance by transporting drugs across the cell membrane into stem cells [10] and contributes to the blood–brain barrier (BBB) [11]. Therefore, the anti-tumor effect of TMZ monotherapy is constrained with limited clinical benefits. Most patients who are given TMZ monotherapy are unable to avoid tumor recurrence after surgery or drug resistance. There is a grave clinical problem that needs to be solved urgently regarding the limitations of TMZ monotherapy for patients with GBM in the absence of a broader range of broad-spectrum and effective chemotherapy drugs.

Levetiracetam (LEV), as an antiepileptic drug (AED), is mainly used for the treatment of partial seizures in adults and children with epilepsy over 4 years of age. In recent years, studies have found that levetiracetam was associated with a better survival rate when used in GBM patients [12–15]. Although these observational studies provide a compelling background for investigating the effect of LEV on GBM, the limitation of these studies stems from retrospective nature, including selection and information bias. On the other side, antiepileptic drugs used asynchronously with chemotherapy might underpower the potency of the anti-tumor effect of AEDs. It is not clear how LEV improves survival in patients with GBM. The potential antitumor activity of LEV might be due to the inhibition of MGMT transcription and expression in glioblastoma cell lines by increasing p53 binding on the MGMT promoter, thus sensitizing glioblastoma cells to the alkylating agent TMZ [16]. In addition, Scicchitano et al. reported that LEV enhances the effect of TMZ on proliferation and apoptosis in GBM cancer stem cells [17]. Furthermore, a study in vitro showed that the tumor-suppressing effect and the induction of cellular senescence of LEV are further enhanced when TMZ is combined with β-galactosidase activity [18]. The combination of TMZ and LEV is expected to offer complementary benefits and thus amplify the anti-tumor effect of TMZ.

Studies have demonstrated that the combination of LEV and TMZ is emerging as an optimal approach for improving the survival of GBM patients after surgery. It is therefore essential to conduct a prospective randomized controlled trial (RCT) to evaluate the effectiveness of TMZ plus LEV compared with TMZ standard chemotherapy. Seventy-three patients were enrolled in a Korean study (NCT02815410) that evaluated the effectiveness of LEV in GBM treatment. No differences were found in survival as a result of LEV. LEV had no significant side effects or overdose symptoms in the study, even when patients received an increased dose of up to 2000 mg/day and other AEDs as well [19]. However, the study was limited by a single-arm study design with external and historical control. In China, there is no correlative, randomized, and controlled trial that has been conducted to investigate the effects of LEV for GBM. As a result, we decided to conduct an RCT to explore the therapeutic effect of LEV plus TMZ for GBM, and thus provide an available therapeutic option for the treatment of GBM. The primary objective of this RCT is to determine whether the combination of TMZ and LEV is superior to standard TMZ chemotherapy in prolonging the progression-free survival (PFS) of GBM patients after surgery. This trial will also evaluate the efficacy of new chemotherapy regimens in improving the overall survival (OS), objective response rate (ORR), and the Karnofsky Performance status scale (KPS).

## Methods/design

### Study design

**Fig. 1:**
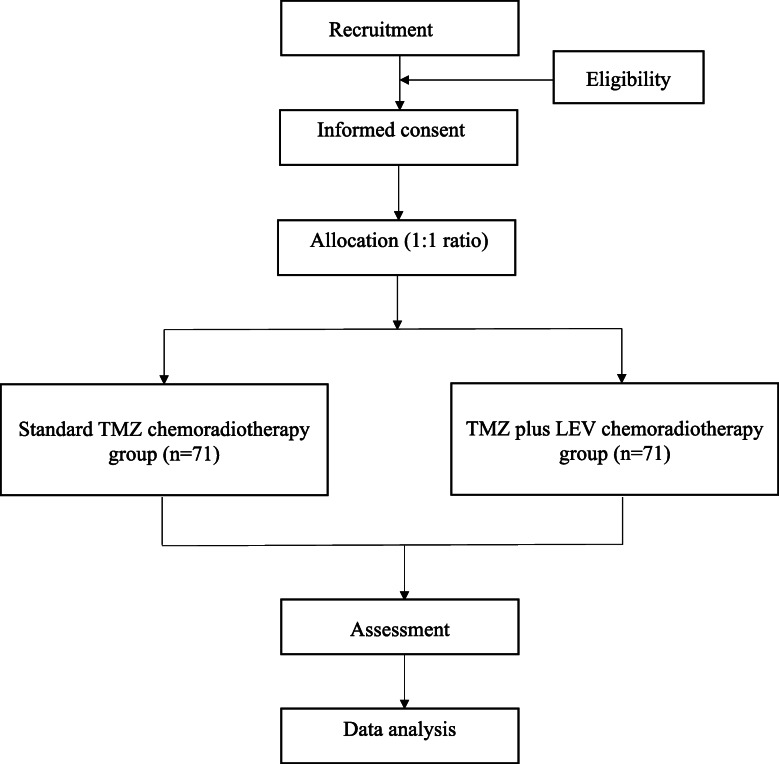
The trial flow chart

This is a prospective, single-center, randomized, double-blind, clinical trial to be conducted at the Second Affiliated Hospital of Chongqing Medical University in China. The study was registered with the Chinese Clinical Trial Registry on 14 August 2021 (ChiCTR-2100049941). The plan is to recruit 142 eligible subjects and assign them to either the control group (standard TMZ chemoradiotherapy) or the experimental group (TMZ plus LEV chemoradiotherapy) at a 1:1 ratio. Participants will undergo a 34-week treatment period and a 3-year follow-up period. The trial flow chart is shown in Fig. 1. The study protocol rigorously follows the Standard Protocol Items: Recommendations for Interventional Trials 2013 (SPIRIT 2013) checklist (see Additional file 1).

### Recruitment and eligibility criteria

The enrollment will be completed within 3 years (from the beginning of recruitment to the last patient). Enrolment is projected to begin in November 2021. Recruitment advertisements for the study will be placed on the WeChat public website and the hospital website. The research staff (NH, YHT, and GJZ) will screen inpatients and outpatients according to the inclusion and exclusion criteria.

The inclusion criteria are as follows:

1. Aged 18–65 years, female or male
2. Newly diagnosed GBM (WHO grade IV) patients with maximal surgical resection
3. Patients within 3 months of surgery
4. KPS ≥50
5. Adequate hematological, renal, and hepatic function. All patients should meet the following criteria: (a) absolute neutrophil count (ANC) ≥ 1.5 × 10^9^/L and platelet count≥100 × 10^9^/L, (b) serum creatinine clearance ≥80 mL/min, (c) total bilirubin level ≤ 1.5 × ULN (except patients with Gilbert syndrome), and (d) aspartate aminotransferase (AST) ≤ 3.0 × ULN, alanine aminotransferase (ALT) ≤ 3.0 × ULN, and AST/ALT < 2.5 × ULN
6. The patient and his/her family members were informed and provided signed and informed consent

The exclusion criteria are as follows:

1. Prior chemotherapy within the last 5 years
2. Planned surgery for other diseases
3. Pregnant women or those that are breastfeeding
4. Patients with a known hypersensitivity to LEV and TMZ or any of the excipients of the products
5. Concurrent illness, including unstable heart disease despite appropriate treatment, a history of myocardial infarction within 6 months, and active hepatitis (hepatitis B virus (HBV) or hepatitis C virus (HCV))
6. Patients with serious psychological disease

The withdrawal criteria are as follows:

1. Voluntary withdrawal during the intervention
2. Alcohol and/or drug abuse
3. Patients who take part in another trial concerning glioma
4. Any other reasons considered inappropriate by the investigators

### Outcomes

2.3.1 Primary outcome

The primary outcome is the PFS of patients, the time from randomization and group allocation to any recorded disease progression, and even death.

2.3.2 Secondary outcomes are as follows:

1. Overall survival (OS), the time from randomization and allocation to death for any reason
2. The Karnofsky Performance status scale (KPS). The scale measures the levels of patient activity and medical needs. The score ranges from 100 (no signs of disease) to 0 (dead).
3. Objective response rate (ORR) = (CR + PR)**/**total number of cases × 100%. A complete response (CR) is defined as the disappearance of all target lesions. A partial response (PR) is defined as a reduction of at least 30% in the sum of diameters of the target lesions, considering the baseline sum diameters as a reference. The ORR will be determined by MRI according to the Response Evaluation Citeria in Solid Tumors (RECIST) [20].
4. Incidence of adverse events (AEs). Adverse events (CTC-Toxicity ≥ grade III) will be recorded and analyzed based on the Common Terminology Criteria for Adverse Events (CTC-AE) [21].

#### Other measures

Complete blood counts (CBC): ANC, platelet count; liver and renal function: serum creatinine clearance, total bilirubin level, aspartate aminotransferase (AST), and alanine aminotransferase (ALT)

### Interventions

The patients will be allocated to either the experimental group or the control group based on a blocked randomization sequence generated by SPSS 11 software for windows. A total of 34 weeks’ worth of medications will be prescribed to each group (including an interval of 4 weeks). The trial protocol was developed in accordance with the latest clinical standard care for GBM [22]. The standard of treatment for GBM patients who are under the age of 70 and have the good physical ability after maximal surgical resection currently consists of chemotherapy and radiation therapy (RT). Therefore, the interventions will be conducted according to the current clinical practice guidelines. A chart showing the administration of interventions is shown in Table 1.

**Table 1:** A chart showing drug administration regimens

Treatment		Groups	
		Experimental group	Control group
**CCRT (6 weeks)**	RT	2.0Gy once daily, 5 days per week	
	TMZ	A daily dose of 75 mg/m^2^	
	LEV	500 mg, twice daily	Placebo tablets
**Adjuvant chemotherapy (6 cycles)**	TMZ	A daily dose of 100–200 mg/m^2^	
	LEV	200–1500 mg, twice daily	Placebo tablets

*Abbreviations*: *1 cycle*, 28 days; *RT*, Radiation therapy, *CCRT* Concurrent chemoradiotherapy

#### Concurrent chemoradiotherapy (CCRT)

##### Radiotherapy

Patients in both study arms will receive the same intensity-modulated radiation therapy (IMRT). The radiation dose will be 60Gy in 30 fractions without changing (2.0Gy once daily, 5 days per week for 6 weeks). The particular volumes of radiation treatment will be defined by clinical target volume (CTV) using MRI scan data.

##### Chemotherapy

During radiation therapy, TMZ will be administered orally to both groups at a dose of 75 mg/m^2^. A continuous use of this product would be permitted for 42 days if the non-hematological toxicity ≤ grade I (except for hair loss, nausea, and vomiting), according to the Common Toxicity Criteria (CTC) [21]. The product will be suspended if the following conditions are met: absolute white blood cell count ≥0.5 × 10^9^/L and < 1.5 × 10^9^/L, platelet count ≥10 × 10^9^/L and < 100 × 10^9^/L, and non-hematological toxicity class II. This product will be terminated if the following conditions occur: absolute white blood cell count < 0.5 × 10^9^/L, platelet counts < 10 × 10^9^/L, and non-hematological toxicity class III or IV.

In addition, patients in the experimental group will also receive LEV tablets for the same period of time. To prevent adverse reactions that may occur early in treatment [23], a lower dose will be administrated. The initial treatment dose is 500 mg twice daily for 42 days without any changes. During treatment, placebo tablets will be given to patients in the control group with the same dosage and appearance as LEV.

#### Adjuvant chemotherapy

Four weeks after the end of the CCRT, six cycles of adjuvant therapy will be performed. Each 28-day cycle will begin with a daily dose of 150 mg/m^2^ of TMZ administered in the experimental group and control group. Starting with the second cycle, the dose of TMZ can be increased to 200 mg/m^2^. TMZ dose will be reduced to 100 mg/m^2^ at a daily dose if any of the following conditions are presented: absolute white blood cell count < 1 × 10^9^/L, platelet counts < 50 × 10^9^/L, and non-hematological toxicity class III. Moreover, this product will be terminated if non-hematological toxicity class III reappears or class IV reappears after the dose is reduced.

Based on the administration of TMZ, the dose of LEV in the experimental group will be adjusted to 1500 mg each time, twice a day, continuous for 6 cycles. The dosage of LEV will be adjusted according to renal function as follows: (1) mild abnormalities (creatinine clearance rate 50–79 mL/min): 750 mg, twice a day; (2) moderate abnormalities (creatinine clearance rate 30–49 mL/min): 500 mg, twice daily; and (3) severe abnormalities (creatinine clearance rate < 30 mL/min): 250 mg, twice daily. Increasing or decreasing the dosage by 500 mg/time every 2–4 weeks, twice daily, is recommended. During treatment, patients in the control group will be given placebo tablets with the same dosage and appearance as LEV.

#### Adherence and concomitant care

Treatment adherence will be monitored by the Pill Counting method: if a drug is scheduled for 100 tablets and 20 tablets have been returned, the compliance is 80%. The adherence of participants will be improved by improving communication and supervision and by providing medication guidance during weekly visits (testing CBC, liver, and kidney function weekly).

Due to surgical trauma and tumor tissue, patients with glioma often have epileptic seizures. To control the occurrence of tumor-related epilepsy, antiepileptic drugs (AEDs) will be administrated in the clinic according to current clinical practice. The current clinical practice protocol includes the use of sodium valproate, the common first-line AED in patients with glioma [24, 25]. All of the medications used during the trial will be documented in a case report form (CRF).

The selection criteria and dose administration for antiepileptic use are as follows:

1. The patient suffered from epilepsy postoperatively, and/or epilepsy preoperatively: Patients will receive 20 mg/kg of sodium valproate once daily, and an EEG will be done 3 months later. The drug can be gradually reduced by 50 mg every 2 weeks if there are no obvious abnormalities.
2. Patients without epilepsy before and after surgery: The patients will take 20 mg/kg of sodium valproate once daily for 2 weeks to prevent the occurrence of epilepsy, then the drugs will gradually be discontinued.
3. Patients experiencing seizures during drug withdrawal: stopping withdrawal immediately and restoring the dosage to pre-seizure level.

### Baseline and follow-up visit**s**

A flow diagram of enrollment, interventions, and assessments for participants (participants timeline) is presented in Table 2.

**Table 2:**
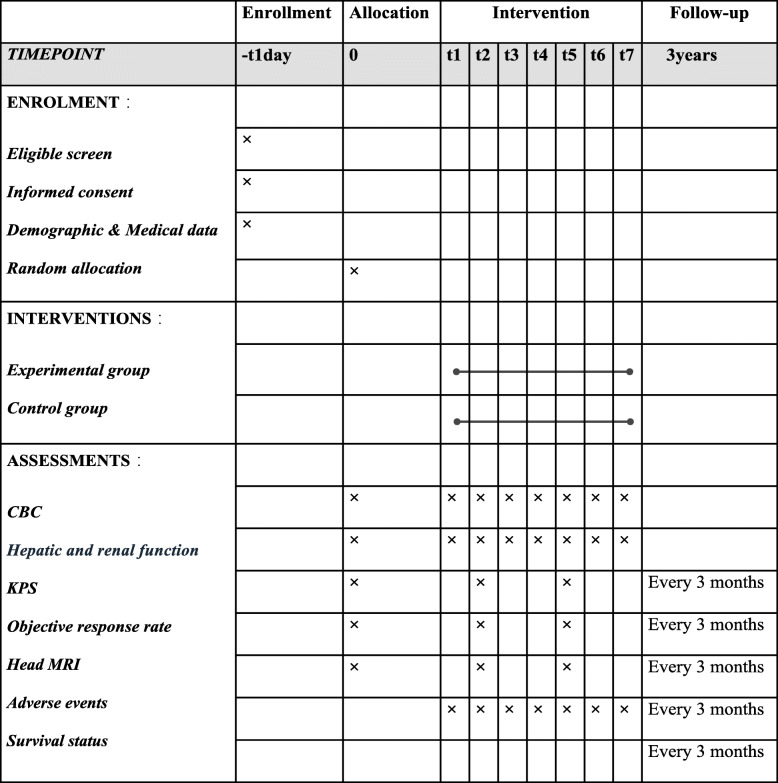
A flow diagram of enrollment, interventions, and assessments for participants

*Abbreviations*: *t1*, 1–6w (concurrent chemoradiotherapy); *t2*, 11–14w; *t3*, 15–18w; *t4*, 19–22w; *t5*, 23–26w; *t6*, 27–30w; *t7*, 31–34w (6 cycles of adjuvant treatment); *CBC*, complete blood counts; *KPS*, Karnofsky Performance Score; *MRI*, magnetic resonance imaging

#### Baseline evaluation: conducted within 2 weeks before the start of the trial

1. Demographic data
2. Past medical history (diagnosis and treatment)
3. KPS
4. Head MRI
5. The molecular prognostic markers: IDH status (IDH-wildtype/IDH-mutant) and MGMTp status (unmethylated/methylated)
6. Laboratory test: complete blood counts (CBC) and liver and renal function

#### During intervention

1. Laboratory test: complete blood counts (CBC) and liver and renal function (monitored weekly)
2. KPS (every 3 months)
3. Head MRI (every 3 months)
4. Objective response rates (every 3 months)
5. Adverse events and concomitant medications

#### Follow-up: every 3 months after treatment

1. KPS
2. Head MRI
3. Objective response rates
4. Adverse events
5. Survival status
6. Any new anticancer treatment(s)

### Sample size

The determination of sample size was calculated by PASS 15.0 software. Based on a previous study and clinical assumptions [26], the median OS of standard care for GBM is 14.6 months while that of the experimental group (TMZ plus LEV ) is 24 months. Statistical parameters are set as follows: one-sided log-rank testing with 80% power, the probability of obtaining a false positive with a statistical test at 0.05, and a 3-year follow-up time. Considering a potential dropout rate of 10% across both groups, it is estimated that 142 patients will need to be enrolled (71 participants per group).

### Randomization and blinding

Eligible subjects will be randomly divided into two parallel pairs of groups in a 1:1 ratio according to a software-generated random sequence (produced using SPSS software). Before study group assignment, the allocation sequence will be concealed using sealed, opaque, and stapled envelopes that will not be opened by participants or recruiters. The implementation of sequence generation and allocation concealment will be implemented by a researcher who will not be involved in the recruitment process. A specific investigator will be responsible for processing study group assignments.

The participants and outcome assessors will remain blind to the group allocation until the end of the trial. The study data statistician will be unaware of allocations and will not participate in the follow-up visit. Regular unblinding will be performed by the principal investigator and statistical experts for the first time to conduct statistical analysis according to the statistical plan. Researchers will perform a second blinding process to determine which of the two groups is the experimental group. To prevent unnecessary unblinding or harm, the emergency unblinding of allocation will be performed if patients meet the following criteria: (1) severe violation of the treatment plan, (2) severe adverse events during the intervention, (3) patients or their family members request to stop the trial, and (4) visitors who are lost in follow-up.

### Data collection and management

A case report form (CRF) will be filled in for each participant before intervention. The collection of baseline data, along with primary and secondary outcomes, will be recorded completely in CRF. Each participant (corresponding to a CRF) will be assigned a specific number by which intervention and follow-up will be performed. Using ResMan, an Internet-based Electronic Data Capture (EDC) tool, all information will be kept independently as double copies in a computer. The data files will be locked after a blind audit and confirmed to be reliable. The CRF shall be owned by the principal investigator and shall not be provided to any third party in any form without the written approval of the principal investigator, who will oversee all of the final trial data. A follow-up registration form will be given to all patients to encourage retention. A researcher will collect the reason for loss, survival condition, and recent outcome assessment for participants who lose from intervention. The original paper files will be kept in the filing cabinet of the sponsoring organization and clinical data will be kept for 5 years.

### Quality control

A data monitoring committee (DMC), which will consist of neurosurgery experts and statisticians, will be established; this will be independent of the sponsor and trial investigator. Periodically, the DMC will assess the progress of the clinical trials, safety data, and important efficacy data. The DMC will also perform an interim evaluation and advise if the trial should be modified or discontinued based on the results of the interim evaluation.

In the course of the study, an inspector will verify the accuracy and completeness of the CRFs and compare them with the source documents. Inspecting must ensure that the subject’s dose changes, treatment changes, adverse events, combined medications, missed visits, and examination omissions are noted in the CRFs. Additionally, they must confirm that the withdrawals and missed visits of the selected subjects have been recorded and explained in the CRFs. As well, the inspector will notify the researcher of the errors, omissions, or unclear handwriting in CRFs and ensure that correction, addition, or deletion is carried out by the researcher or authorized person. The modification must be documented in writing, if necessary. The inspector will promptly inform the principal after each supervision and inspection.

### Statistical analysis

We will use SPSS 23.0 statistical software for Windows to perform statistical analysis. To ensure the reliability of our conclusions, we plan to use intention-to-treat (ITT) analysis as the main form of analysis.

#### Baseline description

Means ± standard deviation (SD) will be used to describe quantitative data that are normally distributed; medians and interquartile ranges (IQRs) will be used to describe non-normal distribution data. The constituent ratio or relative ratio will be used to describe the count data.

#### Comparison of baseline data

*T*-tests will be performed for quantitative data that are normally distributed. A non-parametric test will be used for quantitative data that is not normally distributed. The chi-squared (*χ*^2^) test will be used for counting data. A two-sided *P* value < 0.05 will be considered to be statically significant.

#### Comparison of therapeutic efficacy

A Kaplan–Meier survival analysis with a Log-rank test will be used to compare survival data (PFS and OS) between the two arms. Confounding factors, including demographic data, medical history of drug use prior to intervention, epileptic seizure, and the molecular prognostic markers, will be analyzed by Cox proportion hazards regression analysis. Results will be expressed as hazard ratios (HRs) and 95% confidence intervals (CIs). Furthermore, the KPS and the ORR (ranked data) of the two arms will be compared by Wilcoxon’s rank-sum test.

#### Safety evaluation

Incidence of adverse events (CTC-Toxicity ≥ grade3) will be expressed as the number of patients (percentage) and be analyzed by the chi-square (*χ*^2^) test.

### Reports of adverse events

Adverse events (AEs) will be assessed primarily by abnormal variations in laboratory data (including complete blood counts (CBC), liver function tests (total bilirubin level, AST, ALT), renal function tests (creatinine clearance, blood urea nitrogen), and clinical symptoms. The occurrence, severity level, management strategies, the causality related to the experimental agents, and the termination of all adverse events (AEs) will be recorded and preserved during clinical trials and follow-up. All severe adverse events (CTC-Toxicity ≥ grade3) will be reported promptly to the principal investigator and the research ethics committee (REC). The researchers and clinical trial institutions will ensure that the subjects are treated appropriately and informed truthfully of the relevant information. According to China’s Code of Quality Management for Drug Clinical Trials, researchers are responsible for the cost of dealing with adverse reactions and compensation for patients who suffer adverse reactions. Participants are not provided with specific post-trial care.

## Discussion

In the past, adjuvant chemotherapy for GBM has traditionally been performed with nitrosoureas, such as carmustine (BCNU) and lomustine (CCNU) [27, 28]. Drug resistance and side effects limit the efficacy of these drugs. PCV is a combination of procarbazine, CCBU, and vincristine based on these studies. However, studies have shown that there are no significant survival benefits associated with the use of PCV [29]. Stupp et al. [26] demonstrated in 2005 that concurrent and adjuvant TMZ therapy for 6 months significantly improved the median survival rate for patients with glioblastoma (14.6 months compared to 12.1 months). The Stupp protocol is the most effective chemotherapy to treat GBM to date.

Despite advances in chemotherapy therapy, patients with GBM who have undergone surgery are confronted by drug resistance and tumor recurrence; these factors can lead to immense physical and financial stress. Hwang et al. reported the negative results for patients with GBM in their clinical trial for LEV on the survival benefits of patients with GBM in 2022 [19], which is opposite to their observational study [30]. However, this study was limited by its single-arm design. If our results indicate that the new combination chemotherapy (TMZ plus LEV) is effective, then this clinical trial could provide high-level evidence and broad potential applications for GBM as a first-line treatment regime. Patients with GBM, especially those with TMZ resistance and those with MGMT positivity, will benefit from this new regimen by targeting multiple anti-tumor mechanisms. In China, this is the first prospective, randomized, and controlled clinical trial to investigate the efficacy of TMZ combined with LEV in patients with GBM after surgery. Additionally, we will use PFS as a primary outcome indicator and will also capture several secondary outcome parameters. These relatively objective evaluation indices will be used to evaluate the effectiveness of the new combination regimens.

The study has several limitations that need to be considered. First, while the recruited patients underwent maximum surgical resection, the extent of tumor resection remains a controversial prognostic factor due to the different tumor locations and different surgeons. There is no doubt that, in the treatment of GBM, the role of collaborative drugs cannot take precedence over surgery. Second, since there is no coherent guidance that can be referenced regarding the optimal use of LEV in patients with GBM (for example, treatment period, optimal dose, and discontinuation indicators), the administration of LEV has been a lack of evidence. Previous studies have demonstrated that LEV was generally well tolerated in epilepsy treatment without serious systemic adverse reactions [23, 31]. In the trial, LEV will be administered according to the common dose range for seizure control that has been adopted. Third, since all patients will be recruited from a single hospital, the results of the trial cannot be generalized to other ethnicities and regions. The study is also limited by its relatively small sample size.

In summary, this RCT may contribute to the development of an optimal treatment regimen to extend the survival rate of patients with GBM. Further research is needed to verify the role of LEV in improving the survival of patients with GBM and controlling epilepsy.

## Trial status

This trial has been registered at ClinicalTrials.gov (ID: ChiCTR2100049941). Currently, the study is recruiting patients, and recruitment is expected to finish in November 2024.

The protocol version number and date: version 3, 9 Mar 2022

## Supplementary Information

**Additional file 1.** Standard Protocol Items: Recommendations for Interventional Trials 2013 (SPIRIT 2013) checklist.

## Data Availability

Please contact the corresponding author: 304678@hospital.cqmu.edu.cn.

## Abbreviations

GBM: Glioblastoma
TMZ: Temozolomide
LEV: Levetiracetam
RCT: Randomized controlled trial
OS: Overall survival
PFS: Progress-free survival
KPS: The Karnofsky Performance Status Scale
ORR: Objective response rate
CR: Complete response
PR: Partial response
RT: Radiation therapy
CCRT: Concurrent chemoradiotherapy
BBB: Blood–brain barrier
MGMT: O^6^-Methylguanine methyltransferase
ABC: ATP-binding cassette
ANC: Absolute neutrophil count
ULN: Upper limit of the normal range of each institution
AST: Aspartate aminotransferase
ALT: Alanine aminotransferase
CTV: Clinical target volume
CTC: Common toxicity criteria
RECIST: Respond Evaluation Criteria in Solid Tumors
SPSS: Statistical Product and Service Solutions
AED: Antiepileptic drug
VPA: Valproic acid
CRF: Case report form
EDC: Electronic Data Capture
IQR: Interquartile range
REC: Research ethics committee
AEs: Adverse events
PCV: A combination of procarbazine, CCBU, and vincristine
DMC: Data monitoring committee
